# Differential Modulation of Postprandial Glycemic, Incretin, and Satiety Responses by Low-Digestible Carbohydrates in Humans: An Exploratory Investigation

**DOI:** 10.3390/nu18111742

**Published:** 2026-05-29

**Authors:** Jinsoo Noh, Hye Rim Kim, Jungsook Han, Hwanju Hwang, Jiwon Park, Soonok Sa, Fiona Atkinson, Karen Lau, Sanguine Byun

**Affiliations:** 1 Food R&D, Samyang Corporation, Seongnam 13488, Republic of Korea; jinsoo.noh@samyang.com (J.N.); hyerim.kim1@samyang.com (H.R.K.); jungsook.han@samyang.com (J.H.); hwanju.hwang@samyang.com (H.H.); jiwon.park2@samyang.com (J.P.); soonok.sa@samyang.com (S.S.); 2 Graduate Program in Bioindustrial Engineering, Yonsei University, Seoul 03722, Republic of Korea; 3 Department of Biotechnology, Yonsei University, Seoul 03722, Republic of Korea; 4 School of Life and Environmental Sciences and the Charles Perkins Centre, The University of Sydney, Sydney 2006, NSW, Australia; fiona.atkinson@sydney.edu.au (F.A.); karen.ky.lau@sydney.edu.au (K.L.)

**Keywords:** low-digestible carbohydrates, allulose, 1-kestose, resistant maltodextrin, fructo-oligosaccharides, postprandial glycemia, glycemic control, GLP-1, satiety, functional food, incretin response, insulin dynamics

## Abstract

Background: Effective postprandial glycemic regulation is essential for preventing metabolic disorders such as type 2 diabetes. While pharmacological interventions like GLP-1 (Glucagon-Like Peptide-1) receptor agonists are effective, dietary strategies using low-digestible carbohydrates (LDCs) may offer a sustainable and complementary approach. Methods: Two human physiological investigations were conducted to evaluate the acute metabolic responses to allulose, 1-kestose, resistant maltodextrin (RD), and fructo-oligosaccharide powder (FOP), administered both in isolation and in conjunction with a reference meal (RM). Results: In Study 1, all tested LDCs elicited minimal plasma glucose responses when consumed alone. In Study 2, distinct metabolic benefits were observed depending on the type of LDCs. Allulose exhibited the strongest effects, significantly reducing postprandial glucose and insulin levels while increasing plasma GLP-1 concentrations. 1-Kestose exhibited significantly lower plasma glucose and insulin incremental area under the curve (iAUC) compared to RM alone, indicating improved glycemic regulation. RD significantly enhanced subjective satiety between 30 and 180 min post-consumption. These findings highlight that each LDC exerts unique physiological effects. Conclusions: Collectively, these results demonstrate that acute LDCs consumption distinctly regulates metabolic responses, supporting their application as functional ingredients in targeted nutritional strategies for managing glycemic and metabolic health.

## 1. Introduction

The escalating prevalence of metabolic disorders, such as obesity and type 2 diabetes mellitus, represents a critical and expanding global health challenge. One of the key dietary factors contributing to the development of these conditions is excessive caloric intake. In modern diets, a substantial proportion of total energy intake is derived from refined sugars and high-glycemic-index carbohydrates, which are rapidly digested and absorbed [1,2,3,4,5,6]. High-carbohydrate diets drive significant postprandial glucose and insulin excursions, accelerating the metabolic dysfunction associated with insulin resistance and cardiometabolic disorders [3].

In response to the rising global burden of metabolic dysfunction, the pharmaceutical landscape has evolved rapidly, resulting in sophisticated interventions aimed at restoring glucose homeostasis. Among these, incretin-based therapies targeting the GLP-1 signaling pathway have emerged as a cornerstone of modern treatment. These agents exert multifaceted effects, including the stimulation of glucose-dependent insulin secretion and slowing of gastric emptying to enhance postprandial satiety [7,8,9]. These mechanisms have led to the widespread clinical use of GLP-1 receptor agonists for improving glycemic control and body weight management. However, long-term pharmacological treatment is associated with limitations, including various side effects, high cost, and the need for ongoing administration, highlighting the importance of complementary dietary strategies in metabolic health management. In fact, a Bayesian network meta-analysis of 48 randomized controlled trials (*n* = 27,729) reported an overall gastrointestinal adverse event incidence of 11.66% among GLP-1 RA users, with nausea as the most frequent side effect (21.49%) [10].

These tolerability concerns have renewed interest in non-pharmacological strategies, with dietary modification emerging as a key approach for managing postprandial glycemic regulation and metabolic health in obesity and diet-related disease [11]. To this end, nutritional approaches have increasingly focused on replacing rapidly digestible carbohydrates with dietary fiber and/or low-calorie sweeteners that preserve palatability while attenuating glycemic impact. In this context, LDCs have attracted increasing interest as functional food ingredients. Representative examples include allulose, 1-kestose, resistant maltodextrin, and fructo-oligosaccharides (FOS), which are characterized by limited digestibility and favorable metabolic effects such as attenuated postprandial glucose responses [11] and improved gut-related metabolic functions [12].

While all four LDCs share the common property of limited digestibility, their mechanisms of actions and metabolic effects are distinct. Among these ingredients, allulose, a rare sugar and C-3 epimer of D-fructose, provides negligible calories due to its low-calorie, non-metabolizing nature and has been reported to inhibit α-glucosidase activity, thereby reducing the rate of glucose absorption [13,14]. Specifically, allulose is absorbed in the small intestine through the fructose transporter GLUT5 and the sodium-dependent glucose cotransporter SGLT1 [15]. Unlike conventional sugars, absorbed allulose is not metabolized and is excreted unchanged in the urine [16]. In contrast, 1-kestose (a trisaccharide component of FOS, Short-chain FOS, scFOS) and RD are recognized for their prebiotic properties and their ability to modulate gut-derived signals related to satiety and glucose metabolism [17,18]. These metabolic benefits are largely mediated through the gut–metabolism axis, whereby the gastrointestinal tract communicates bidirectionally with peripheral metabolic organs via neural, hormonal, and microbial signals [19]. More specifically, upon reaching the distal intestine, fermentable LDCs modulate the gut microbiota, promoting the secretion of short-chain fatty acids (SCFAs) such as butyrate, propionate, and acetate [20]. These SCFAs act as signaling molecules for G-protein-coupled receptors (GPR41 and GPR43) expressed on enteroendocrine L-cells, thereby stimulating the secretion of GLP-1 and PYY [21]—hormones which are well studied for regulating postprandial glucose secretion, gastric emptying, and appetite regulation [22].

While the individual metabolic profiles of these ingredients have been studied in isolation, there is limited clinical evidence regarding their interactive effects when consumed alongside standard meals, which more closely reflect real-world dietary patterns. Specifically, whether LDCs can stimulate endogenous GLP-1 secretion—and thereby mimic the metabolic benefits associated with pharmacological incretin-based therapies—remains an open and clinically relevant question. Addressing this gap may provide evidence-based support for dietary strategies that complement or reduce reliance on pharmacological interventions.

In this study, we conducted two human physiological investigations to systematically evaluate the metabolic efficacy of these functional ingredients. Study 1 established the baseline relative glycemic response of allulose, 1-kestose, and RD when consumed alone to confirm their low-glycemic nature. Study 2 further investigated how these ingredients modulate postprandial glucose, insulin, and GLP-1 responses, as well as subjective satiety, when consumed with a standardized rice meal in individuals with elevated fasting glucose levels. Overall, this study aims to contribute to the scientific understanding of LDCs as functional ingredients in food formulations designed to manage metabolic health.

## 2. Materials and Methods

### 2.1. Study Participants and Ethics

A reference food was administered during the first and last test sessions, while the order of the test ingredients administered between the reference sessions was randomized across participants. Two distinct human physiological investigations were conducted at Sydney University’s Glycemic Index Research Service (SUGiRS), Australia. For Study 1, ten healthy, non-smoking adults (5 males and 5 females) were recruited, with a mean age of 27.4 ± 8.3 years and a mean BMI of 22.1 ± 1.9 kg/m^2^. For Study 2, a total of 10 healthy, nonsmoking adults with elevated fasting blood glucose concentrations ranging from 5.6 to 6.1 mmol/L were recruited. Eligible participants were aged between 18 and 65 years. Exclusion criteria included being overweight or underweight, currently dieting, suffering from any illness or food allergy, or regularly taking prescription medication, with the exception of standard contraceptive medication. The study sample comprised six males and four females, with a mean age of 39.4 years (range: 24.6–55.8 years). The mean body mass index (BMI) was 22.7 kg/m^2^ (range: 19.2–24.6 kg/m^2^), indicating that all participants fell within the healthy weight range as defined by a BMI of 18.5–24.9 kg/m^2^. Both studies were approved by the Human Research Ethics Committee of the University of Sydney (Approval No. 2021/782, 11 November 2021) and were conducted in accordance with the Declaration of Helsinki.

### 2.2. Test Materials and Methodology

The glycemic index was determined according to the internationally recognized methodology (ISO 26642:2010) [23] at SUGiRS. This methodology has been validated for its reproducibility and clinical utility in ranking carbohydrate-containing foods [11,12]. Due to the lack of available carbohydrates in the test products, the standardized methodology was modified to use 25-g total carbohydrate portions. The test materials provided by Samyang Corporation (Seongnam, Republic of Korea) included allulose (Nexweet^®^), 1-kestose, resistant maltodextrin (RD; Fiberest^®^ HF), and fructo-oligosaccharides (FOS). Study 1 (Isolated Intake): Participants consumed portions containing 25 g of total carbohydrate (e.g., 25.1 g allulose, 25.4 g 1-kestose) dissolved in 250 g of water. A 25 g available carbohydrate glucose solution served as the control. Study 2 (Mixed Meal): Participants consumed a standardized high-glycemic reference meal (RM; steamed Jasmine rice, peas, and soy sauce) providing 75 g of available carbohydrate (Table 1). In test sessions, 10 g of each functional ingredient was dissolved in 250 g of water and consumed alongside the RM.

### 2.3. Human Study Protocol and Biochemical Analysis

Both studies were conducted as Human Physiological Investigations, with sessions separated by a minimum one-day washout period. Both studies were exploratory in nature, and all analyses should be interpreted accordingly. Participants were instructed to consume a carbohydrate-rich evening meal (excluding legumes) the night before each test session, followed by an overnight fast of at least 10 h. They were also required to avoid alcohol and unusual levels of food intake and physical activity on the day prior to each session and reported to the research center the following morning in a fasting state. Participants consumed the assigned test or reference food within 12 min. Sampling: In Study 1, capillary blood samples were collected at 0, 15, 30, 45, 60, 90, and 120 min. In Study 2, the monitoring period was extended to 180 min to assess plasma insulin and GLP-1. Active GLP-1 was measured using a validated commercial multiplex immunoassay with internal standards and controls (Milliplex^®^ Human Metabolic Hormone Panel V3, Merck Healthcare Pty Ltd., Macquarie Park, NSW, Australia), performed by an external laboratory (Cardinal Bioresearch Pty Ltd., Slacks Creek, QLD, Australia). DPP-IV inhibitors were added at the time of blood collection to preserve active GLP-1 integrity, and samples were stored at −80 °C prior to analysis. Subjective satiety was assessed at each time point using a Rating Scale (RS units) and was evaluated over the 0–180 min. Plasma glucose was measured using a glucose hexokinase enzymatic assay on a Beckman Coulter AU480 analyzer (Beckman Coulter, Brea, CA, USA).

### 2.4. Statistical Analysis

Data is presented as the mean ± standard error (SEM) unless otherwise indicated. Statistical analyses were performed using GraphPad Prism version 11 (GraphPad Software, San Diego, CA, USA). The iAUC for glucose and insulin was calculated using the trapezoidal method, and differences between groups were analyzed using the Wilcoxon matched-pairs signed-rank test. Changes in glucose and insulin levels over time were analyzed using a linear mixed-effects model to account for repeated measurements within subjects and to evaluate the effects of time, group, and their interaction. A *p*-value < 0.05 was considered statistically significant.

## 3. Results

### 3.1. Study Design

**Figure 1:**
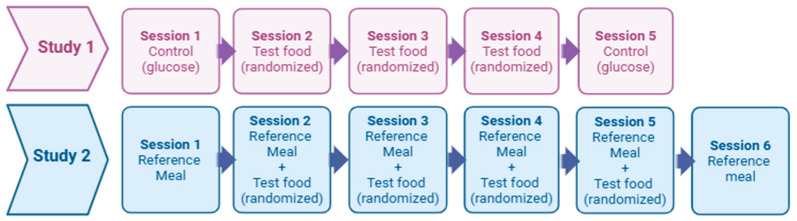
Schematic overview of the designs for Study 1 and Study 2.

A schematic overview of the study designs is presented in Figure 1. Two separate studies were conducted to evaluate the effects of LDCs on glycemic and incretin responses. In the first study, three LDCs ingredients (allulose, 1-kestose, and RD) were administered individually, and postprandial glycemic responses were monitored for 120 min, using glucose as the control. In the second study, four LDCs ingredients (allulose, 1-kestose, RD, FOP) were co-administered with a reference meal (RM) to evaluate their effects on postprandial plasma glucose, insulin, GLP-1 concentrations, and subjective satiety.

In Study 1, participants completed five test sessions: they consumed a glucose solution (control) on two separate occasions and three LDCs test foods (allulose, 1-kestose, and RD) on one occasion each, in randomized order. In Study 2, participants completed six test sessions: they consumed a reference meal alone on two occasions, and the reference meal combined with one of four LDCs test foods (allulose, 1-kestose, RD, and FOP) on four separate randomized occasions. All sessions were conducted in the morning following an overnight fast.

### 3.2. Effects of LDCs Ingredients on Blood Glucose Responses (Study 1)

**Figure 2:**
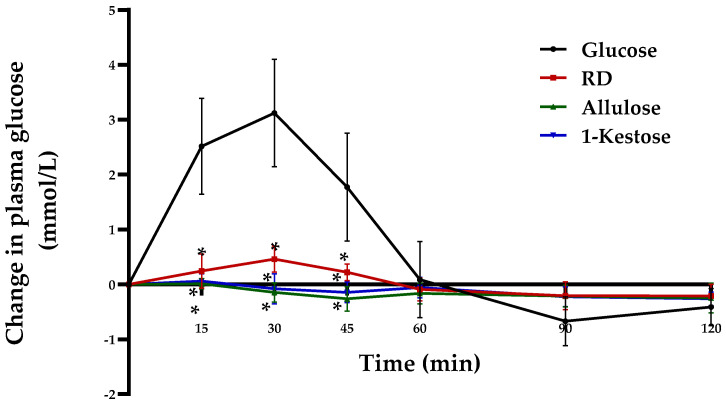
Plasma glucose response curves for Study 1. Comparing the control (glucose solution, black) and the three LDC foods—RD (red), 1-kestose (blue), and allulose (green)—expressed as the change in plasma glucose concentration from baseline. Error bars represent the standard error of the mean (SEM). * *p* < 0.05 vs. glucose. RD = Resistant Maltodextrin.

In Study 1, the acute glucose responses to three LDCs ingredients (allulose, 1-kestose, and RD), each containing 25 g of total carbohydrates, were measured over 120 min (Figure 2). The glucose control produced the expected rapid increase in plasma glucose concentrations, peaking at approximately 30 min. In contrast, all three test foods showed significantly lower plasma glucose levels compared to the glucose control (*p* < 0.05). Specifically, 1-kestose and allulose elicited minimal glucose responses, remaining close to baseline throughout the 120-min period. RD produced a slightly greater increment in plasma glucose compared with allulose and 1-kestose, although the response remained significantly lower than that of the glucose control. Overall, these findings demonstrate that allulose, 1-kestose, and RD did not elicit a statistically significant increase in plasma glucose levels when consumed alone, with concentrations maintaining near-baseline values.

### 3.3. Attenuation of Postprandial Glucose and Insulin Responses by LDCs (Study 2)

**Figure 3:**
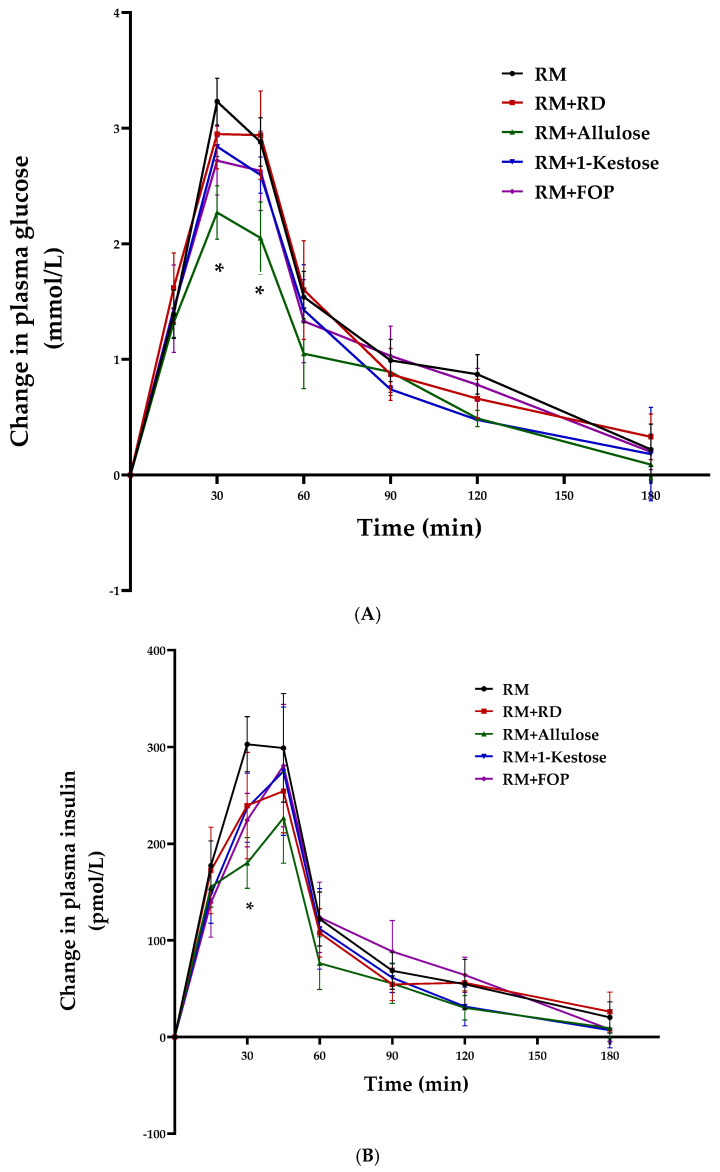

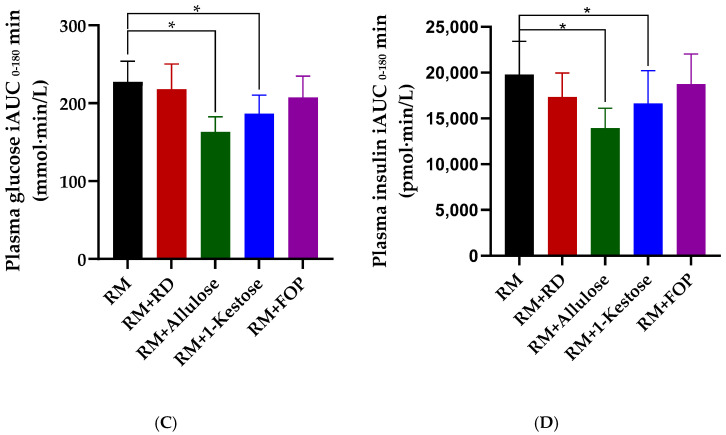
Plasma glucose and insulin responses in Study 2 following consumption of RM alone or together with LDCs. (**A**) Average plasma glucose response curves expressed as change from baseline for RM (black), RM with resistant maltodextrin (RM + RD, red), allulose (RM + allulose, green), 1-kestose (RM + 1-kestose, blue), and FOP (RM + FOP, purple). (**B**) Average plasma insulin response curves over 180 min expressed as change from baseline for the same treatments. (**C**) iAUC (iAUC_0–180_ min) for plasma glucose. (**D**) iAUC (iAUC_0–180_ min) for plasma insulin. Values are presented as mean ± standard error of the mean (SEM). * *p* < 0.05 vs. RM. LDCs = Low-Digestible Carbohydrates; RM = Reference Meal; RD = Resistant Maltodextrin; FOP = Fructo-Oligosaccharide Powder.

Study 2 assessed the postprandial plasma glucose and insulin responses following consumption of an RM with or without LDCs foods (i.e., RD, allulose, 1-kestose, and FOP) (Figure 3A–D). Plasma glucose concentrations increased rapidly after ingestion of the RM, reaching a peak after approximately 30 min (Δ3.23 ± 0.20 mmol/L), followed by a gradual decline toward baseline over the 180-min period. Compared with RM alone, co-consumption of allulose significantly attenuated the postprandial plasma glucose response (Figure 3A). More specifically, allulose significantly reduced the plasma glucose response at 30 min (Δ2.27 ± 0.23 mmol/L, *p* = 0.0061) and 45 min (Δ2.05 ± 0.31 mmol/L, *p* = 0.0257), consistent with its known mechanism of competitively occupying the binding sites of intestinal glucose transporters SGLT1 and GLUT2, thereby reducing glucose absorption in the small intestine [15]. In contrast, RD, 1-kestose, and FOP showed a trend toward a lower glucose response but did not significantly differ from the RM control.

The plasma insulin responses following consumption of RM with or without LDCs foods are shown in Figure 3B. The administration of RM alone resulted in a marked elevation of plasma insulin concentrations. In contrast, the co-consumption of allulose significantly attenuated this response. Specifically, at 30 min, allulose was found to significantly reduce insulin levels to Δ180 ± 26 pmol/L (*p* = 0.0416), whereas the consumption of RM alone reached a peak of Δ302 ± 28 pmol/L, which is consistent with the well-established glucose-dependent nature of insulin secretion—whereby a lower glycemic stimulus results in a proportionally reduced insulinemic response [24]. In contrast, RD, 1-kestose and FOP showed a trend toward lower insulin responses compared with the RM control but did not differ significantly.

The iAUC for plasma glucose is shown in Figure 3C. The glucose iAUC was significantly reduced for RM + allulose (163 ± 19 mmol·min /L, p = 0.0029) and RM + 1-kestose (186 ± 23 mmol·min /L, p = 0.0195), whereas RM + RD (217 ± 32 mmol·min /L) and RM + FOP (207 ± 27 mmol·min /L) did not differ significantly from the RM control (227 ± 26 mmol·min /L), although their values were lower than the RM control.

The iAUC for plasma insulin is presented in Figure 3D. The insulin iAUC was significantly reduced for RM + allulose (13,956 ± 2163 pmol·min /L, *p* = 0.0029) and RM + 1-kestose (16,642 ± 3582 pmol·min/L, *p* = 0.0098), whereas RM + RD (17,319 ± 2646 pmol·min/L) and RM + FOP (18,747 ± 3284 pmol·min/L) did not differ significantly from the RM control (19,797 ± 3636 pmol·min /L). The reduction in insulin iAUC observed with allulose and 1-kestose is consistent with the reduction in postprandial glucose iAUC (Figure 3C), reinforcing the glucose-dependent mechanism of insulin secretion in response to LDCs co-consumption.

### 3.4. Effects of LDCs on Postprandial GLP-1 Responses with a Reference Meal (Study 2)

**Figure 4:**
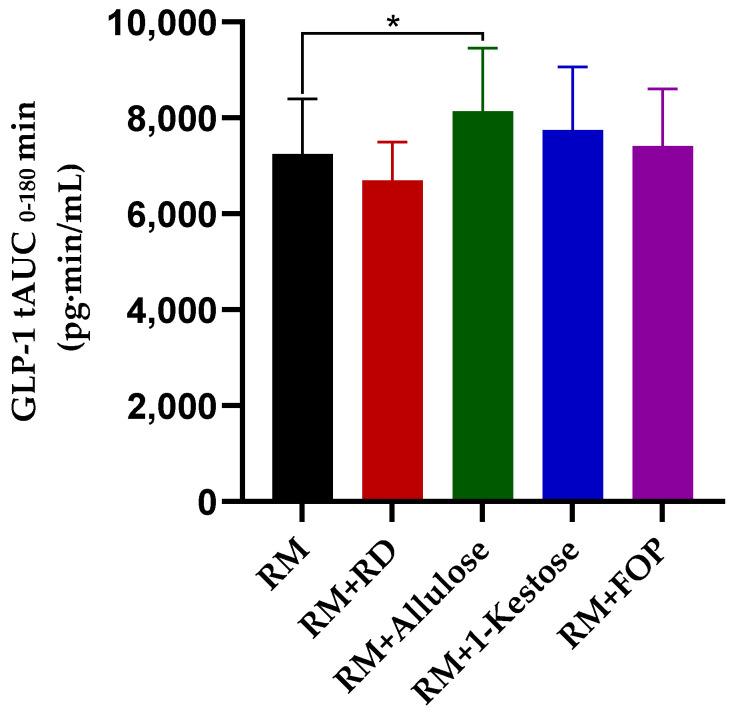
Plasma GLP-1 levels following consumption of RM alone or together with LDCs. tAUC for plasma GLP-1 (pg·min/mL) over 180 min in Study 2, comparing the reference meal (RM, black) and four LDCs foods—Resistant maltodextrin (RM + RD, red), allulose (RM + allulose, green), 1-kestose (RM + 1-kestose, blue), and FOP (RM + FOP, purple). Bars represent mean ± standard error of the mean (SEM). * *p* < 0.05 vs. RM. LDCs = Low-Digestible Carbohydrates; RM = Reference Meal; RD = Resistant Maltodextrin; FOP = Fructo-Oligosaccharide Powder.

The total AUC (tAUC) of plasma GLP-1 following the consumption of RM with or without LDC foods is presented in Figure 4. Among the tested ingredients, RM + allulose showed a significantly higher GLP-1 tAUC (8136 ± 1319 pg·min/mL, *p* = 0.042) compared with the RM control (7244 ± 1151 pg·min/mL). Notably, the elevated GLP-1 tAUC observed with allulose co-consumption may plausibly contribute to the significant reduction in postprandial glucose iAUC, as GLP-1 is well established to potentiate glucose-dependent insulin secretion, thereby attenuating the postprandial glycemic response [25]. The consequent reduction in postprandial glycemic stimulus would in turn explain the significantly lower insulin iAUC observed with allulose [8]. On the other hand, RD, 1-kestose, and FOP did not significantly increase GLP-1 levels.

### 3.5. Effects of LDCs on Postprandial Satiety Responses with a Reference Meal (Study 2)

**Figure 5:**
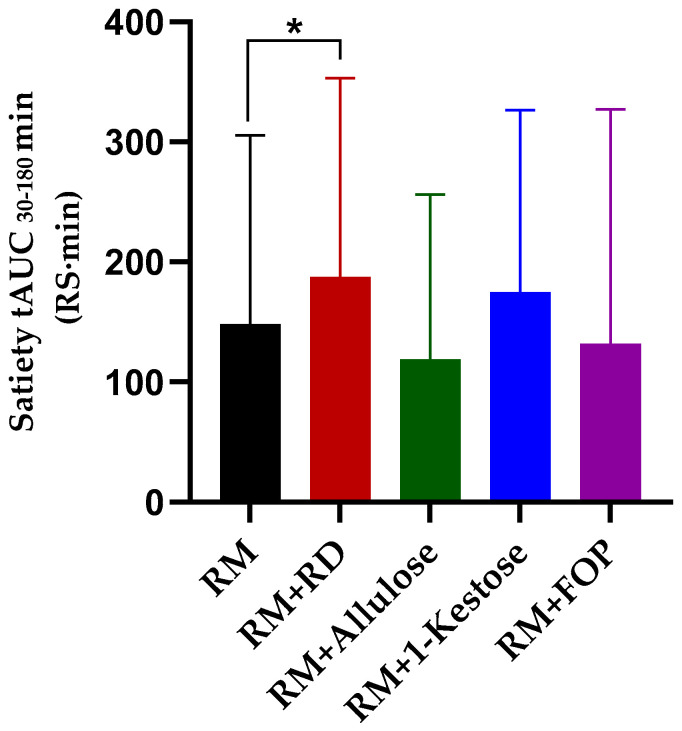
Subjective satiety responses measured in RS (Rating Scale) units, expressed as total AUC (tAUC; RS·min) from 30 to 180 min in Study 2. The reference meal (RM, black) was compared with four LDC foods: Resistant maltodextrin (RM + RD, red), allulose (RM + Allulose, green), 1-kestose (RM + 1-kestose, blue), and FOP (RM + FOP, purple). Values are presented as mean ± SEM. * *p* < 0.05 vs. RM. LDCs = Low-Digestible Carbohydrates; RM = Reference Meal; RD = Resistant Maltodextrin; FOP = Fructo-Oligosaccharide Powder.

Subjective satiety was assessed from 30 to 180 min, and the tAUC is presented in Figure 5. RM + RD showed a significantly higher satiety tAUC (*p* = 0.042) compared with RM alone, indicating an enhanced postprandial satiety response. While RM + 1-kestose elicited a higher satiety tAUC compared to RM alone, this increase did not reach statistical significance.

## 4. Discussion

This study investigated the acute glycemic effects of several LDCs ingredients—allulose, 1-kestose, RD, and FOP—when consumed alone or with a reference meal. When consumed alone, allulose, 1-kestose, and RD elicited minimal rises in plasma glucose, with levels remaining close to baseline. The glucose control produced the expected rapid increase in plasma glucose concentrations, peaking at approximately 30 min, reflecting rapid intestinal absorption of glucose. Such a glycemic spike is characteristic of rapidly digestible carbohydrates and is consistent with previous reports demonstrating that plasma glucose levels in healthy individuals typically reach maximum concentrations within 30 min following ingestion [13,14]. These findings are further supported by recent systematic reviews and meta-analyses showing that rapidly digestible carbohydrates consistently produce significant postprandial glycemic excursions in both healthy and metabolically compromised individuals [26,27].

In contrast, all three test foods showed significantly lower plasma glucose levels compared to the glucose control. Specifically, 1-kestose and allulose elicited minimal glucose response, remaining close to baseline throughout the 120-min period. This may be attributed to the limited intestinal absorption of 1-kestose [28], resulting in a negligible contribution to circulating glucose levels. Furthermore, allulose, a non-metabolizable isomer of fructose, competitively occupies the binding sites of SGLT1 and GLUT2, thereby reducing glucose absorption [15,29]. Consequently, the absorbed fraction is not recognized by glycolytic enzymes and is largely excreted unchanged in the urine, preventing any significant elevation in blood glucose [16], consistent with recent human studies confirming the negligible glycemic impact of allulose across a range of doses [30]. RD produced a slightly greater increment in plasma glucose compared with allulose and 1-kestose, although the response remained significantly lower than that of the glucose control. This may reflect the partial digestibility of RD [31]. Overall, these findings indicate that allulose, 1-kestose, and RD do not produce a meaningful increase in plasma glucose levels when consumed alone, with glucose concentrations remaining close to baseline. This lack of glycemic impact supports their potential as functional carbohydrate alternatives for populations requiring glucose management.

When co-consumed with a reference meal, the LDCs ingredients demonstrated varying degrees of glycemic modulation. Among them, allulose significantly reduced the plasma glucose concentrations at different time points compared with RM alone. This observation is consistent with its well-established mechanism of competitively occupying intestinal glucose transporter binding sites, thereby limiting the rate of glucose absorption in the small intestine [15,32]. In this study, RD did not significantly reduce the plasma glucose response compared with RM alone. This finding contrasts with previous reports indicating that the consumption of RD with a meal can reduce postprandial glucose levels [33]. The discrepancy may be attributed to differences in the composition and structure of the reference meal, which can influence gastric emptying and digestion, thereby influencing the postprandial glycemic response. It is also plausible that the non-significant group-level observation of RD reflects the inherent interindividual variability in metabolic responses, whereby differences in insulin sensitivity and gastrointestinal transit time, compounded by the small sample size of the present study, may have obscured a meaningful glycemic attenuation that could become more apparent in larger cohort studies. Consistent with the postprandial glucose levels, allulose also significantly attenuated the plasma insulin concentrations compared to RM alone.

To further evaluate the cumulative glycemic impact of LDCs ingredients, the iAUC was analyzed for both plasma glucose and insulin. Similarly to the time-course analysis, allulose exhibited significantly lower plasma glucose and insulin iAUC compared to those of RM alone. The significant inhibition of insulin iAUC observed in parallel is likely a direct consequence of the reduced glycemic stimulus, consistent with the glucose-dependent nature of insulin secretion [25], rather than a direct insulinotropic effect of allulose itself. While 1-kestose did not show significant differences at individual time points, it resulted in a significantly lower plasma glucose and insulin iAUC compared to RM alone. This may suggest a more gradual and sustained attenuation of postprandial glycemia rather than an acute inhibitory effect. This pattern is mechanistically distinct from allulose and may reflect a subtle modulation of intestinal transit time. This observed reduction in iAUC is consistent with previous findings [34], where 1-kestose supplementation was shown to improve glucose tolerance and lower cumulative glycemic exposure.

To investigate the potential mechanism of actions of LDCs on glycemic response, plasma GLP-1 levels were measured following the consumption of LDCs with RM. Among the tested ingredients, RM + allulose showed a significantly higher GLP-1 tAUC compared with the RM control. This may be attributed to the ability of allulose to directly stimulate enteroendocrine L-cells in the intestine, thereby promoting GLP-1 secretion and increasing circulating GLP-1 levels [35]. This discrepancy is noteworthy because the elevated GLP-1 tAUC observed with allulose may plausibly contribute to the significant reduction in postprandial glucose iAUC, as GLP-1 is well known to regulate insulin secretion, hence attenuating the postprandial glycemic response [36]. The consequent reduction in glucose iAUC would in turn explain the significantly lower insulin iAUC, consistent with the glucose-dependent nature of insulin secretion. It is important to note that the diet-induced GLP-1 elevation observed with allulose reflects a physiological response and should not be equated with the sustained supraphysiological receptor activation achieved through pharmacological GLP-1-based therapies. On the other hand, RD, 1-kestose, and FOP did not significantly increase GLP-1 levels. RD [37] and oligosaccharides [38] have been reported to increase GLP-1 secretion primarily through fermentation by the gut microbiota, which produces SCFAs that stimulate L-cells in the colon. Since the present study evaluated acute postprandial responses, the lack of significant GLP-1 elevation for 1-kestose, FOP, and resistant maltodextrin may reflect the requirement for prolonged consumption to allow sufficient microbial metabolism and SCFAs accumulation to reach levels capable of meaningfully stimulating GLP-1 secretion.

Beyond assessing glycemic and insulinemic responses, it is important to evaluate the impacts of LDCs ingredients on postprandial satiety, as these metabolic markers play an essential role in appetite regulation. Subjective satiety was analyzed from 30 to 180 min rather than from a 0-to-180-min window, as gastric emptying research indicates that by 30 min, a significant portion of the meal has transited from the stomach into the small intestine to trigger hormonal feedback [39]. It was shown that RD exhibited significantly higher satiety tAUC compared to RM alone. This may be attributed to its physicochemical properties as a soluble dietary fiber. RD has been shown to possess a high water-holding capacity, which may increase gastric distension and slow gastric emptying, thereby enhancing satiety signals [40]. In contrast, 1-kestose and FOP did not significantly increase satiety. As prebiotic dietary fibers, their effects are mediated through microbial fermentation and SCFA production, which requires longer-term intake to promote satiety. Interestingly, although allulose significantly increased serum GLP-1 levels, this did not translate into a significant increase in subjective satiety. One plausible explanation is that the magnitude of endogenous GLP-1 secretion induced by allulose may have been insufficient to influence perceived satiety [7]. In addition, satiety is regulated by multiple gut hormones and neural signals, including PYY, CCK, and ghrelin, suggesting that GLP-1 alone may be insufficient to significantly influence satiety responses [41].

Taken together, the findings reveal a differential but complementary metabolic modulation among the four LDCs compounds tested. Allulose was the most metabolically active in the acute postprandial setting, significantly attenuating glucose and insulin responses while simultaneously elevating GLP-1 tAUC, suggesting that its effects are mediated through both inhibition of intestinal glucose absorption and enhanced incretin signaling. 1-Kestose demonstrated a more modest but sustained effect, evidenced by significantly reduced glucose and insulin iAUC in the absence of significant GLP-1 elevation, suggesting an alternative mechanism possibly involving subtle modulation of intestinal transit time. Importantly, RD was the only compound to significantly enhance subjective satiety in the acute setting, possibly attributable to its physicochemical properties as a soluble fiber rather than hormonal mechanisms. Collectively, these findings highlight that LDC compounds exert their metabolic effects through distinct yet complementary mechanisms, suggesting that their combined use in functional food formulations may offer additive benefits in postprandial metabolic regulation. Furthermore, no adverse events or gastrointestinal symptoms were reported by any participant in either Study 1 or Study 2.

Several limitations of the present study should be acknowledged. The small sample size (*n* = 10 per study) limits statistical power and generalizability, reflecting the pilot nature of this investigation. Additionally, the single-blind design may introduce a degree of bias, which should be considered when interpreting the findings. Furthermore, interindividual variability in insulin sensitivity, incretin secretory capacity, and gastrointestinal transit time may have contributed to the heterogeneous metabolic responses observed, particularly the non-significant trends seen with RD and FOP, and should be considered when interpreting the results. Therefore, larger, double-blind, randomized controlled trials with subgroup analyses stratified by metabolic phenotype and more comprehensive outcome measures are warranted to validate and extend the findings of the present study.

Collectively, these findings suggest that the acute consumption of LDCs ingredients may beneficially influence postprandial metabolic responses through multiple physiological pathways. However, as several additional metabolic effects of carbohydrate ingredients are mediated through gut microbiota-dependent mechanisms, longer-term studies are warranted to further investigate their impact on gut microbiota modulation and subsequent metabolic health outcomes.

## 5. Conclusions

In conclusion, this pilot study demonstrates that LDCs—allulose, 1-kestose, RD, and FOP—exert distinct and differential effects on acute postprandial metabolic responses when consumed alone or with a reference meal. Specifically, allulose significantly reduced postprandial glucose and insulin responses while elevating GLP-1 levels, 1-kestose improved cumulative glycemic control, and RD significantly enhanced subjective satiety. These findings highlight the potential of LDCs ingredients as functional dietary components targeting postprandial glycemic and metabolic regulation. Building upon these preliminary findings, future larger-scale, double-blind, randomized controlled trials incorporating longer intervention periods and more comprehensive mechanistic outcome measures are required to further establish the prolonged metabolic benefits and clinical applicability of LDCs ingredients in dietary strategies for glycemic and metabolic health management.

## Figures and Tables

**Table 1:** Composition of the RM and test meals (RM + LDCs), including portion size, energy, macronutrient, available carbohydrate, sugar, and fiber content. Values were calculated using manufacturers’ data. LDCs = Low-Digestible Carbohydrates; RM = Reference Meal; RD = Resistant Maltodextrin; FOP = Fructo-Oligosaccharide Powder.

Meal	Portion Size (g)	Energy (kJ)	Protein (g)	Fat (g)	Available Carbohydrates (g)	Sugar (g)	Fiber (g)
RM	249.9 g	1646	10.5	2.9	77.8 *	3.5	4.5
RM + RD	249.9 g + 10 g	1730	10.5	2.9	77.8 *	3.9	13.5
RM + Allulose	249.9 g + 10 g	1663	10.5	2.9	77.8 *	3.5	4.5
RM + 1-Kestose	249.9 g + 10 g	1730	10.5	2.9	77.8 *	3.6	14.5
RM + FOP	249.9 g + 10 g	1734	10.5	2.9	77.8 *	3.9	14.0

* A small amount of available carbohydrate (2.8 g) was provided by the green peas + soy sauce.

## Data Availability

All relevant data from this study are provided within the article. For any additional information, please contact the corresponding author.

## Abbreviations

The following abbreviations are used in this manuscript:

LDCs	Low-digestible carbohydrates
RM	Reference meal
GLP-1	Glucagon-like peptide-1
iAUC	Incremental area under the curve
tAUC	Total area under the curve
RD	Resistant maltodextrin
FOS	Fructo-oligosaccharides
FOP	Fructo-oligosaccharide powder
SUGiRS	Sydney University Glycemic Index Research Service
SEM	Standard error of the mean
